# Epileptic Networks in Focal Cortical Dysplasia Revealed Using Electroencephalography–Functional Magnetic Resonance Imaging

**DOI:** 10.1002/ana.22535

**Published:** 2011-12-07

**Authors:** Rachel Thornton, Serge Vulliemoz, Roman Rodionov, David W Carmichael, Umair J Chaudhary, Beate Diehl, Helmut Laufs, Christian Vollmar, Andrew W McEvoy, Matthew C Walker, Fabrice Bartolomei, Maxime Guye, Patrick Chauvel, John S Duncan, Louis Lemieux

**Affiliations:** 1 Department of Clinical and Experimental Epilepsy, University College London Institute of NeurologyLondon, United Kingdom; 2 Epilepsy Society Magnetic Resonance Imaging Unit, Chalfont St PeterBuckinghamshire, United Kingdom; 3 Neurophysiology and Neuropsychology Laboratory, French Institute of Health and Medical ResearchMarseille, France; 4 Faculty of Medicine, University of the MediterraneanMarseille, France; 5 CHU Timone, Neurophysiology Clinic Service, Public Assistance of the Hospitals of MarseilleMarseille, France; 6 Biological and Medical Magnetic Resonance CenterMarseille, France

## Abstract

**Objective:**

Surgical treatment of focal epilepsy in patients with focal cortical dysplasia (FCD) is most successful if all epileptogenic tissue is resected. This may not be evident on structural magnetic resonance imaging (MRI), so intracranial electroencephalography (icEEG) is needed to delineate the seizure onset zone (SOZ). EEG-functional MRI (fMRI) can reveal interictal discharge (IED)-related hemodynamic changes in the irritative zone (IZ). We assessed the value of EEG-fMRI in patients with FCD-associated focal epilepsy by examining the relationship between IED-related hemodynamic changes, icEEG findings, and postoperative outcome.

**Methods:**

Twenty-three patients with FCD-associated focal epilepsy undergoing presurgical evaluation including icEEG underwent simultaneous EEG-fMRI at 3T. IED-related hemodynamic changes were modeled, and results were overlaid on coregistered T1-weighted MRI scans fused with computed tomography scans showing the intracranial electrodes. IED-related hemodynamic changes were compared with the SOZ on icEEG and postoperative outcome at 1 year.

**Results:**

Twelve of 23 patients had IEDs during recording, and 11 of 12 had significant IED-related hemodynamic changes. The fMRI results were concordant with the SOZ in 5 of 11 patients, all of whom had a solitary SOZ on icEEG. Four of 5 had >50% reduction in seizure frequency following resective surgery. The remaining 6 of 11 patients had widespread or discordant regions of IED-related fMRI signal change. Five of 6 had either a poor surgical outcome (<50% reduction in seizure frequency) or widespread SOZ precluding surgery.

**Interpretation:**

Comparison of EEG-fMRI with icEEG suggests that EEG-fMRI may provide useful additional information about the SOZ in FCD. Widely distributed discordant regions of IED-related hemodynamic change appear to be associated with a widespread SOZ and poor postsurgical outcome. ANN NEUROL 2011

Focal cortical dysplasia (FCD) results from abnormal neuronal migration and is commonly associated with pharmacoresistant focal epilepsy.^1^ Surgical treatment often requires intracranial electroencephalography (icEEG) recordings to localize the seizure onset zone (SOZ) and map eloquent cortex, and has the best outcome when all epileptogenic tissue is resected.^2^ Recently the classification of these abnormalities has been revisited, dividing them into 3 subgroups based on clinicopathological features.^3^ Type 2B, in particular, is frequently seen on structural magnetic resonance imaging (MRI), but in a significant number, current structural MRI appears normal.^4^, ^5^ icEEG studies have challenged the idea that epilepsy in FCD is associated with a solitary epileptogenic lesion, and report discrete dysplastic foci and additional, remote, structurally normal, epileptogenic areas of cortex.^6^, ^7^ The presence of these epileptogenic areas remote from the primary dysplastic lesion is reported to be associated with poorer outcome. There is therefore a need for better detection and evaluation of the involvement of these distributed epileptogenic foci and their potential impact on SOZ localization and surgical efficacy in patients with FCD.

EEG-functional MRI (fMRI) recordings have been used to study cerebral neural activity associated with interictal discharges (IEDs),^8^ by measurement of hemodynamic changes (blood oxygenation level-dependent [BOLD] contrast^9^) and there is increasing interest in the technique's clinical potential.^10^, ^11^ Studies have demonstrated areas of IED-related BOLD signal change concordant with the putative seizure onset zone in 50 to 70% of patients in whom IEDs are recorded,^12^, ^13^ but they frequently show distributed patterns involving regions remote from the presumed focus, some of which may represent regions of seizure or IED propagation.^14^ Case reports and small series using EEG-fMRI in patients with FCD show that clusters of IED-correlated BOLD contrast were both local to and remote from the SOZ, including subcortical structures.^15^–^17^

Comparisons between IED-correlated fMRI results and icEEG, considered the gold standard technique for localization of epileptic foci, have generally been limited to case descriptions in the context of studies of patients with epilepsy of mixed etiology.^12^, ^13^ More systematic studies consist of 2 case series,^11^, ^18^ the most comprehensive of which demonstrated that electrodes within the vicinity of an EEG-fMRI peak usually include at least 1 active contact.^18^ We sought to build on these findings in the light of increasing evidence that FCD contributes to an epileptic network rather than forming a discrete epileptic focus, which has implications for successful surgical treatment.^19^

We aimed to prospectively compare presurgical IED-related BOLD signal changes with the results of icEEG and postoperative outcome in patients with FCD. We also aimed to assess whether regions of IED-related BOLD signal change relate to epileptogenic regions in FCD, which would have potential implications for surgical efficacy where multiple regions of signal change were detected.

## Patients and Methods

### Subjects

Sixty-five patients with refractory focal epilepsy undergoing presurgical evaluation and awaiting icEEG, from 4 centers (National Hospital for Neurology and Neurosurgery, London, UK; Kings College Hospital, London, UK; Frenchay Hospital, North Bristol NHS Trust, UK; and CHU La Timone, Marseille, France), underwent EEG-fMRI at the Epilepsy Society MRI Unit, Chalfont St Peter, United Kingdom. Twenty-three had a diagnosis of FCD made on structural MRI, histology, or both and were selected for analysis. All procedures were subject to the relevant local and national research ethics committees' approval.

### Electroclinical Evaluation

Patients had scalp EEG video-telemetry, MRI, and in some cases positron emission tomography (PET) and/or single photon emission computed tomography (SPECT) according to their local treatment center protocol. All underwent structural MRI at the Epilepsy Society MRI Unit, according to the Epilepsy Society protocol, at 3T.^19^ They subsequently underwent icEEG with a tailored electrode implantation, determined by the clinical team at the patient's center using subdural grids, depth electrodes, or both, followed by surgical resection if appropriate. The EEG-fMRI results were not used in the planning of icEEG or resections that were undertaken with curative intent. Postoperative outcome was recorded at 12 months using the International League Against Epilepsy scale (ILAE).^20^ Surgical outcome was considered good for a reduction in seizure days of ≥50% (ILAE 1-4) and poor for a reduction of <50% or an increase in seizure days (ILAE 5-6).

### icEEG Analysis

The SOZ was identified on icEEG by experienced observers and described by the electrode(s) at which low-amplitude fast activity was first seen at seizure onset. The irritative zone (IZ) was defined as the region giving rise to IEDs on icEEG (described by the electrodes where IEDs were recorded). Regions to which the seizure onset rhythms rapidly (<3 seconds) propagated (following the observation that resection of regions in which seizure onset rhythms are seen within 3 seconds is associated with good outcome^21^) were noted. The term *multifocal SOZ* refers to situations where seizure onset was recorded at several discrete sites, as opposed to propagation of seizure activity from a solitary SOZ.

### EEG-fMRI Acquisition and Preprocessing

Thirty-two or 64 EEG channels were recorded using a commercial magnetic resonance (MR)-compatible system (BrainAmp MR and Brain Vision Analyzer; Brain Products GMbH, Munich, Germany). Further details can be found in our previous publications.^14^, ^23^ Resting EEG was recorded for 5 to 20 minutes prior to scanning sessions, and resting state EEG-fMRI was recorded for two or three 20-minute sessions as tolerated. Sessions consisted of 404 T2*-weighted single-shot gradient-echo echo-planar images (EPIs; echo time/repetition time, 30/3000 milliseconds; flip angle, 90°; 43 2.5mm interleaved slices; FOV, 24 × 24cm^2^; matrix, 64 × 64) acquired continuously on a 3T Signa Excite HDX MRI scanner (General Electric, Milwaukee, WI). EPI time series were realigned and spatially smoothed with a cubic Gaussian Kernel of 8mm full width at half maximum.

### EEG Preprocessing and Analysis

MR gradient and pulse-related artifact were removed from the EEG^23^, ^24^ using a commercial EEG processing package (Brain Analyzer; Brain Products), and IEDs were marked.

### fMRI Analysis

fMRI time series data were analyzed using a general linear model to determine the presence of regional IED-related BOLD changes in SPM5 (spm.fil.ac.uk). For this purpose, IEDs were represented as zero-duration events (unit impulse δ functions), and convolved with the canonical hemodynamic response function and its temporal and dispersion derivatives, resulting in 3 regressors for each event type.^25^ Further details of the analysis can be found in our previous publication.^22^ Motion and cardiac pulse-related effects were modeled as confounds.^25^, ^26^ Effects of interest were tested and mapped using an SPM{F} test and considered significant at a threshold of *p* = 0.05 (family-wise error [FWE] correction for multiple comparisons), but effects seen at a lower level of significance (*p* < 0.001, uncorrected for multiple comparisons) were also reported. The sign of the BOLD change for each cluster was determined by plotting the fitted response at the most significant voxel within the cluster.

### Comparison of the EEG-fMRI Results with Intracranial EEG

Individual T1-weighted MRI scans were coregistered and fused with a postimplantation computed tomography (CT) scan acquired with the icEEG electrodes in situ^28^ and the SPM{F} to identify regions of IED-related BOLD signal change in relation to the icEEG. The locations of the icEEG electrodes and BOLD clusters were verified at operation and by inspection of the EPI data, respectively. The degree of concordance of the EEG-fMRI results with the SOZ was assessed based on the entire statistical maps and summarized as either:

- Concordant (C): All IED-related BOLD signal clusters colocalized (within 2cm and in the same lobe) with the SOZ as identified on icEEG.
- Concordant Plus (C+): Some clusters of significant IED-related BOLD signal change were localized with the SOZ. All other significant BOLD clusters were within the same lobe or touching the edge of the same lobe as the SOZ (to allow for potential coregistration errors).
- Discordant Plus (D+): Some clusters of significant IED-related BOLD signal change were localized within the SOZ, with other significant BOLD clusters in other lobes.
- Discordant (D): All clusters of IED-related BOLD signal change were remote from the SOZ.
- Null: There was no cluster of significant IED-related BOLD change.

## Results

**FIGURE 1:**
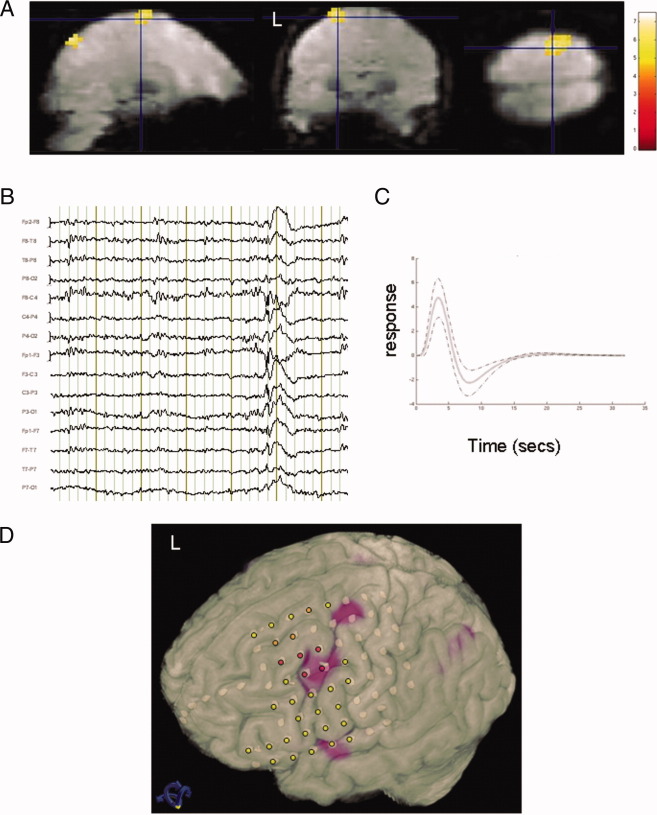
Results of electroencephalographic (EEG) functional magnetic resonance imaging (MRI) and intracranial EEG in a 28-year old female (Patient 3) with normal structural MRI and focal epilepsy (age of onset, 12 years). EEG revealed left (L) frontal spike wave complexes (maximal, F3). The patient underwent intracranial recording with a 48-contact grid placed over the lateral convexity of the left frontal lobe and a 16-contact grid placed over the left temporal lobe, following which she underwent resection of the abnormality in its entirety and had a significant reduction in seizure frequency (International League Against Epilepsy scale class 3) 12 months after surgery. (A) Interictal discharge (IED)-related blood oxygen level-dependent signal (BOLD) activation is overlaid on high-resolution echo planar imaging (family-wise error-corrected for multiple comparisons; *p* < 0.05, *z* = 7.10, crosshair at global maximum). (B) Scalp EEG shows events recorded during scanning. (C) The hemodynamic response related to the events in B is shown. (D) IED-related BOLD increase (magenta) overlaid (magenta) on a surface rendering of the patient's T1-weighted MRI fused with computed tomography taken with intracranial electrodes in situ, showing the relationship of the BOLD change to the seizure onset zone (electrodes depicted in red), areas of rapid seizure propagation (electrodes depicted in orange), and irritative zone (depicted in yellow). The electrodes in the seizure onset zone are concordant with the cluster of BOLD increase containing the global statistical maximum. [Color figure can be viewed in the online issue, which is available at www.annalsofneurology.org.]

**FIGURE 2:**
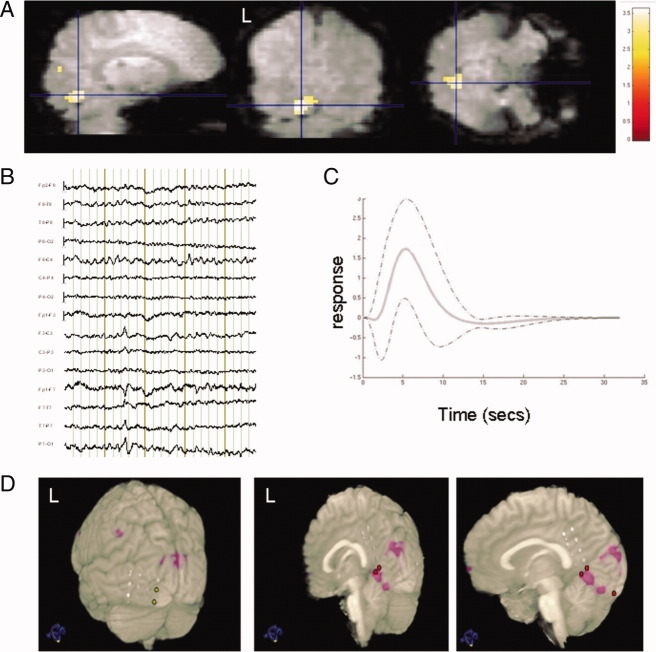
Results of electroencephalographic (EEG) functional magnetic resonance imaging (MRI) and intracranial EEG in a 21-year-old male patient (Patient 22) with normal structural MRI and focal epilepsy (age of onset, 9 years). EEG revealed left (L) frontotemporal spikes and sharp waves (maximal, F3–F7). Seizures consisted of a visual aura followed by a sensation of the eyes pulling to the right. The patient underwent intracranial recording with a 20-contact lateral occipital grid and 16-contact mesial occipital grid in addition to 2 × 6-contact occipital depth electrodes, following which he underwent surgical resection of the seizure onset zone, close to the left occipital pole, following which he was seizure free (International League Against Epilepsy scale outcome 1) 1 year after surgery. (A) Interictal discharge (IED)-correlated blood oxygen level-dependent signal (BOLD) signal change is overlaid on T2*-weighted echo planar imaging (SPM{F}: *z* = 3.87, *p* < 0.05 family-wise error corrected for multiple comparisons). (B) Scalp EEG shows events recorded during scanning. (C) The hemodynamic response related to the events in B is shown. (D) IED-related BOLD increase (magenta) overlaid (magenta) on a surface and volume rendering of patient T1-weighted MRI fused with computed tomography taken with intracranial electrodes in situ, showing the relationship of the BOLD change to the seizure onset zone (electrodes depicted in red), and irritative zone (depicted in yellow). The electrodes in the seizure onset zone are concordant with the cluster of BOLD increase containing the global statistical maximum. Note that volume rendered images show a cross section through the left medial occipital lobe. [Color figure can be viewed in the online issue, which is available at www.annalsofneurology.org.]

IEDs were recorded during EEG-fMRI scanning in 12 of 23 cases, of whom 11 had statistically significant IED-related BOLD changes. Tables 1, 2, and 3: Summarize the clinical data, EEG-fMRI results and icEEG results, respectively. Representative cases are illustrated (Figs 1 and 2 and Supplementary Figs 1–4). 3T MRI revealed FCD in 9 of 11 cases. Structural MRI was normal in cases 3 and 22 (both C+).

**Table 1:** Clinical Details of Cases

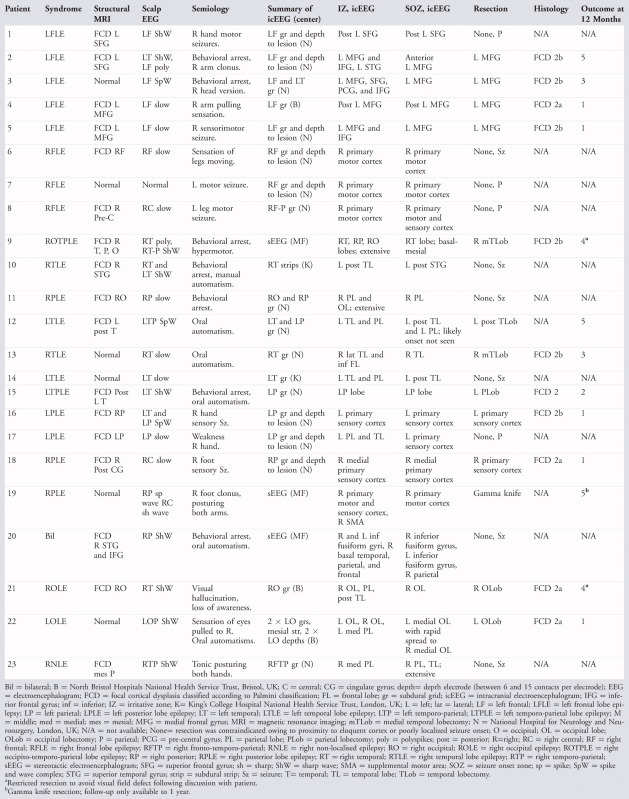

**Table 2:** EEG-fMRI and icEEG Results in Cases with IED during EEG-fMRI

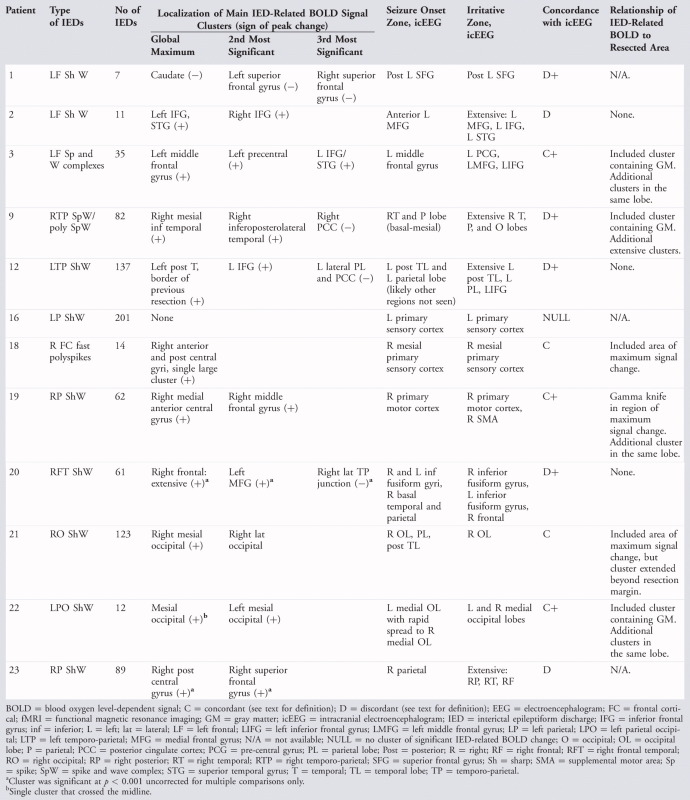

**Table 3:** Details of Intracranial EEG Recordings and Surgical Resections in Cases with IED during EEG-Fmri

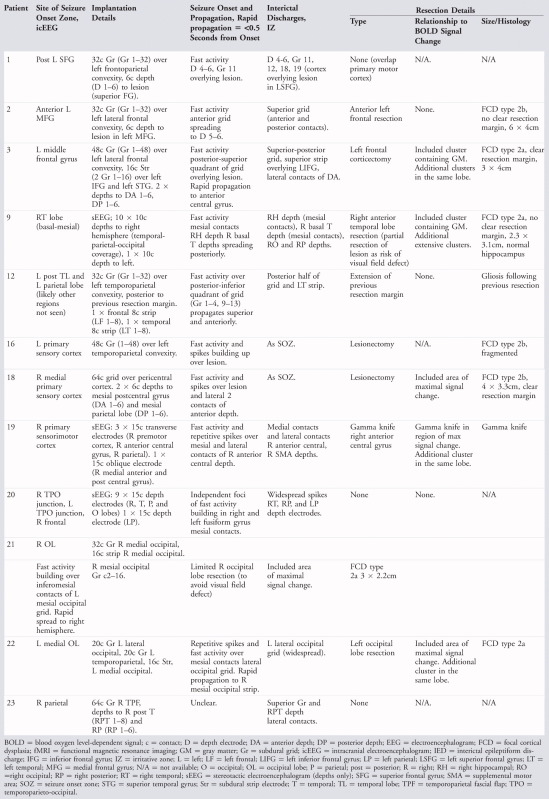

### Relationship of IED-Related BOLD Signal Change to icEEG

Ten of 11 patients had IED-related BOLD increases, of whom 2 were classified C (Patients 18 and 21), 3 were classified C+ (Patients 3 [Fig 1], 19, and 22 [Fig 2]), 3 were classified D+ (Patients 9, 12, and 20), and 2 were classified D (Patients 2 and 23). In Patient 1, only BOLD signal decreases were observed (classified D+). BOLD decreases were also observed in Patients 9, 12, and 20.

### Patients with C Results

Patient 18 had a single cluster of IED-related BOLD signal change, the global statistical maximum of which colocalized with the SOZ. The cluster also colocalized with electrodes to which seizure activity rapidly propagated and the IZ. Patient 21 had a single cluster of BOLD signal change colocalized with the SOZ. The IZ extended beyond the regions of BOLD signal change.

### Patients with C+ Results

In addition to the clusters colocalized with the SOZ, BOLD clusters colocalized with electrodes to which seizure activity rapidly propagated in 2 of 3 patients (3 and 22); in Patient 19, clusters lay outside the region of rapid seizure propagation, but colocalized with the IZ.

### Patients with D+ Results

Three of 4 patients (9, 12, and 20) had clusters of BOLD increase colocalized with the IZ in addition to clusters colocalized with the SOZ. In Patient 1, all IED-associated BOLD changes were decreases, 1 of which colocalized with the SOZ and IZ. In all 4 patients, there were IED-related BOLD clusters outside the icEEG coverage.

### Patients with D Results

In Patient 2, a single BOLD cluster colocalized in part with the IZ, which was extensive on icEEG, but remote from the SOZ. In Patient 23, a single BOLD cluster was revealed deep in the (widespread) IZ but remote from the SOZ, which was presumed to lie outside the region covered by icEEG.

### Surgical Outcome and Relationship of IED-Related BOLD Signal Change to Area of Resection

Of the patients classified C, Patient 18 was seizure free 12 months after resection (ILAE 1), and Patient 21 had a >50% reduction in seizure frequency (ILAE 4), following a resection that was limited because of the risk of a visual field deficit. The surgical resection included the region of most significant BOLD increase in both patients.

Two of the 3 patients classified C+ (Patients 3 and 22) had a good outcome (ILAE 3 and 1, respectively), and the resection included the cluster of BOLD signal increase colocalized with the SOZ. In the remaining patient (Patient 19), a single seizure focus was identified around the central sulcus, and the patient underwent gamma knife surgery with ILAE outcome of 5 at 1 year.

Of the patients classified D+, Patients 9 and 12 had a poor outcome (ILAE class 5 or 6), and there were regions of significant BOLD signal increase remote from the resection area. Two of 4 did not undergo resection: Patient 1, who had a highly localized single seizure focus, owing to overlap with primary motor cortex; and Patient 20, due to multiple independent sites of seizure onset.

One patient classified D (Patient 2) had a poor outcome (ILAE class 5) following surgical resection with no overlap with the EEG-fMRI result. The remaining patient (Patient 23), who had IED-related BOLD change remote from the SOZ, did not undergo resection owing to a diffuse SOZ.

#### Patients with No IEDs during EEG-fMRI

Six of 11 patients with no recorded IEDs had a good outcome (ILAE 1-4) following resection of a solitary focus of seizure onset on icEEG, whereas in the remaining 5 patients, resection was precluded or a poor outcome was recorded.

## Discussion

We report the first prospective systematic evaluation of the potential role of EEG-fMRI in the presurgical evaluation of patients with FCD by systematic comparison with icEEG.

EEG-fMRI revealed significant IED-related BOLD clusters in 11 of 12 cases in whom IED were recorded (12 of 23 patients). Comparison with icEEG and consideration of the surgical outcome suggests that in addition to the presence of IED-related clusters within the SOZ, the extent of clusters remote from the SOZ may also be important.

In 9 of 11 patients (classified C, C+, or D+), at least 1 cluster colocalized with the SOZ, and all clusters colocalized with the SOZ at a lobar level in 5 of these 9. Two of 5 had normal structural MRI, and a cluster concordant with a solitary seizure focus, not previously identified during noninvasive evaluation, was found in all 5. These results are comparable with previous EEG-fMRI studies of mixed pathologies and nonlesional cases in focal epilepsy.^10^, ^11^ Six of the 11 FCD patients with no recorded IEDs had a good seizure outcome (ILAE 1-4) following surgical resection.

When multiple IED-related BOLD clusters of positive IED-related BOLD signal change were identified remote from and particularly contralateral to the region of FCD, multiple or diffuse epileptic foci were usually seen on icEEG, precluding successful resective surgery.

### Neurophysiological Significance

#### Relationship to Recent Studies

Our results support evidence from icEEG studies that FCD may be associated with multiple areas of epileptogenicity,^19^, ^29^ some of which are structurally normal, and corroborate EEG-fMRI studies demonstrating that IED-related BOLD signal changes are observed at the same site and remote from the putative seizure onset zone in malformations of cortical development.^15^, ^16^

The observation of IED-related regions of BOLD signal change remote from the seizure onset zone, but concordant with the IZ or regions of rapid propagation on icEEG supports electrospray ionization (ESI) studies, which suggest that EEG-fMRI may be a useful tool to image the epileptic network.^14^, ^28^

#### Sign of BOLD Signal Change

In 1 patient (Patient 1), the IED-related BOLD signal cluster that colocalized with the SOZ was classified as a decrease, consistent with previous studies reporting regions of IED-related BOLD decrease in the IZ or SOZ in a only a small proportion of cases.^16^ Although IED-related BOLD signal decrease in the default mode network is commonly reported^29^, ^30^ and thought to reflect downstream effects, the neurophysiological basis of BOLD signal decrease within the SOZ remains unclear. Recent work has suggested that some of these decreases may reflect the undershoot of a pre-event BOLD signal increase,^31^ or disruption of neurovascular coupling^32^ (this may be especially relevant in FCD, in which γ-aminobutyric acid-mediated synaptic inhibition may be disrupted with a potential impact on coupling).^33^ There were no obvious electroclinical features specific to Patient 1.

### Clinical Significance

It has been suggested that noninvasive localization techniques such as scalp EEG-fMRI could reduce the need for invasive tests such as icEEG, which remains the gold standard for the localization of epileptic foci, but has limited spatial sampling, and is both expensive and not without risk. Although EEG-fMRI is unable to provide the same information as icEEG, it benefits from relatively uniform whole-brain coverage. The finding that multiple widespread IED-related BOLD signal clusters were apparently associated with a widespread SOZ or multiple sites of seizure onset suggests a potential use for EEG-fMRI in determining which patients are likely to benefit from icEEG and those in whom results are likely to be poor.

The size of the group studied here precludes statistically meaningful calculations of sensitivity and specificity with regard to postsurgical outcome. It is of note that in those classified C or C+, surgical outcome was good (ILAE class 1-4) in the majority of cases, whereas surgical outcome was poor (or resection was precluded) in 5 of 6 (83%) of the cases classified D or D+. It should be noted that of those patients classified C or C+, the patients who had the poorest outcomes (ILAE class 5, Patient 19 and ILAE class 4, Patient 21) underwent modified procedures despite a solitary SOZ (gamma knife in Patient 19, meaning 1 year may be too early to assess outcome, and limited resection in Patient 21) to avoid functional deficit. This may explain in part why only 2 of 5 of this group were completely seizure free (ILAE class 1) following resection.

Six of the 11 FCD patients who did not have IED in the EEG-fMRI study had a good seizure outcome. It is not known what BOLD activations would have been found if IED had occurred during these studies. It is evident that the occurrence of IED during EEG-fMRI is not in itself a predictive factor for outcome. What is evident is that in those with FCD, the finding of widespread BOLD activations with IED appears to be associated with widespread epileptic abnormalities and may be a useful factor to include in the decision to undertake invasive EEG studies. EEG-fMRI, however, with the very limited temporal sampling possible, cannot replace icEEG in identifying a target for resection.

The finding of IED-related BOLD changes in patients with normal structural MRI is not new,^10^, ^12^, ^34^ but in both MR-negative cases in whom IEDs were recorded in this series (Patients 3 and 22), localization was concordant and surgical outcome was good, providing further evidence of the potential value of EEG-fMRI in the presurgical evaluation of this group.

There was no relationship between the extent of IED-related BOLD changes and histological subtype, although the majority of the patients had FCD type 2.

#### Relationship to Other Noninvasive Modalities

EEG-fMRI is among several evolving techniques including magnetoencephalography (MEG), ESI (used to inform EEG-fMRI), and isotope imaging used in the noninvasive evaluation of epilepsy. MEG appears to have a higher predictive value for surgical outcome and better sensitivity for SOZ localization than PET and SPECT.^35^, ^36^ In a study of MEG in 27 children with FCD, spike sources were detected in all of those with type 2, of whom 46% had clusters concordant with the SOZ.^37^ Complete resection of clusters was associated with seizure freedom, but of those with scattered sources, 44% were also seizure free, in contrast to regions of widespread IED-related BOLD signal change, which were usually associated with a poor outcome in our data. Comparison with our small sample suggests that MEG is more sensitive than EEG-fMRI, although when IEDs are recorded in EEG-fMRI, concordant clusters are found in a similar proportion of patients. EEG-fMRI may provide more information about the extent of epileptic networks than ESI based on a single equivalent dipole model. Comparative data are required to clarify the roles of each technique, which may provide complementary information.

### Methodological Considerations

#### Limits of an Interictal Study

Due to the difficulty of studying seizures using fMRI, EEG-fMRI typically focuses on IEDs, whereas the gold standard is the SOZ identified on ictal icEEG, a mismatch common to many noninvasive presurgical localization tests^35^; however, there is evidence from functional imaging studies that brain regions responsible for IEDs often closely match the seizure onset and epileptogenic zones.^23^, ^38^, ^39^ Changes in brain state between the EEG-fMRI study and icEEG recording have to be assumed to be minimal to allow comparisons to be made. Although unlikely to become common practice, ictal EEG-fMRI has produced interesting results.^15^, ^40^, ^41^

#### Limits of the Validation

The difficulties in validating EEG-fMRI results, which may reveal multifocal and widespread patterns, are similar to those encountered in other tomographic mapping techniques such as PET and ictal SPECT, and are generally more complex than those in MEG/EEG-based localization, particularly for single-dipole source models. Validation requires summarizing the correspondence between whole-brain maps and the SOZ on icEEG, expressed as concordance. Features of the fMRI maps used for the assessment of concordance include the amplitude, size, and sign of observed BOLD signal change. The concordance scheme used in this study is modified from our previous studies^12^ following a recent comparison of EEG-fMRI and EEG source analysis, showing that in some cases small and less significant clusters can be the most concordant with the IZ,^14^ while reflecting the need for EEG-fMRI result description schemes that are clinically relevant.

The comparison of EEG-fMRI and icEEG is also limited by differences between the signals that can lead to decoupling of hemodynamic and electrophysiological effects over scales on the order of 1cm.^42^ icEEG has restricted spatial sampling, particularly if only depth electrodes, which record from a volume of approximately 1cm^3^ around individual contacts, are used,^43^ whereas subdural grids do not sample directly from neuronal sources and are subject to the inverse problem, similar to scalp EEG.^44^ We cannot, therefore, comment on regions of BOLD signal change that did not lie in the vicinity of an electrode, and given these limitations, we considered that the use of an 8mm smoothing kernel in the fMRI data would not significantly compromise resolution.

#### Coregistration

Coregistration, particularly between EPI and T1-weighted MRI, is limited by EPI signal dropout effects at the brain–cerebrospinal fluid–air interfaces, and intraoperative cortical shifts during electrode placement, estimated by some authors to be up to 24mm.^45^ FCD may not always lie on the cortical convexity, but deep within a sulcus, resulting in further potential inaccuracies when inferring a quantitative relationship between the SOZ and clusters of BOLD signal change.^28^ We therefore limit ourselves to an anatomical description of the IED-related BOLD cluster and the SOZ and an allowance of 2cm to account for displacement in particular.

#### Yield

EEG-fMRI relies on the recording of IEDs during the scanning period. Events were captured in only 52% of this group, as we deliberately included all patients regardless of their resting EEG to avoid selection. Recent developments promise to increase the technique's sensitivity, particularly by using information derived from routine EEG.^14^, ^46^ We reported results that are uncorrected for multiple comparisons but still statistically significant, as we were comparing with the gold standard. Inclusion of confounding factors such as motion and physiological noise in the fMRI model is intended to ensure these are not considered as effects of interest.

### Conclusions

Our results add to the increasing body of evidence that FCD may be multifocal, with areas of epileptogenic tissue remote from the dysplastic lesion in some cases. We found that EEG-fMRI may be useful in identifying those patients who are more likely to have multiple epileptic foci and are therefore less likely to benefit from icEEG and resective surgery, although larger groups and longer outcome data are required to refine the role of EEG-fMRI in the presurgical evaluation of FCD.
