# Supplementary figures and images for: Epileptic Networks in Focal Cortical Dysplasia Revealed Using Electroencephalography–Functional Magnetic Resonance Imaging
Source: Ann Neurol. 2011 Dec 7;70(5):822–37. doi: 10.1002/ana.22535 (PMC3500670; doi:10.1002/ana.22535)

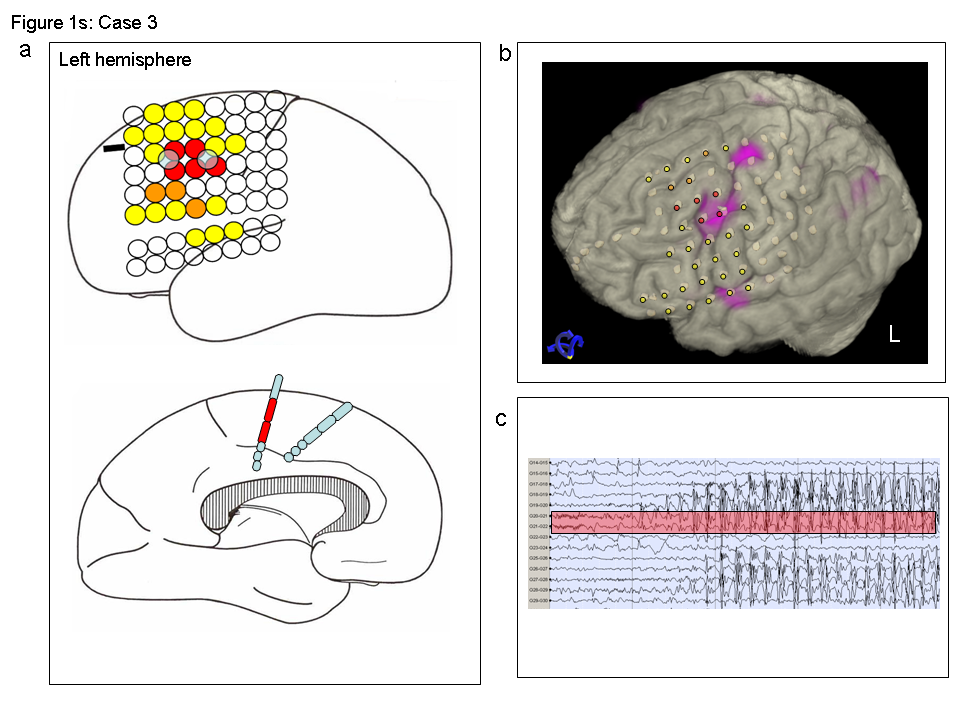

Supplement: Supplementary file 1 [file ana0070-0822-SD1.tif]

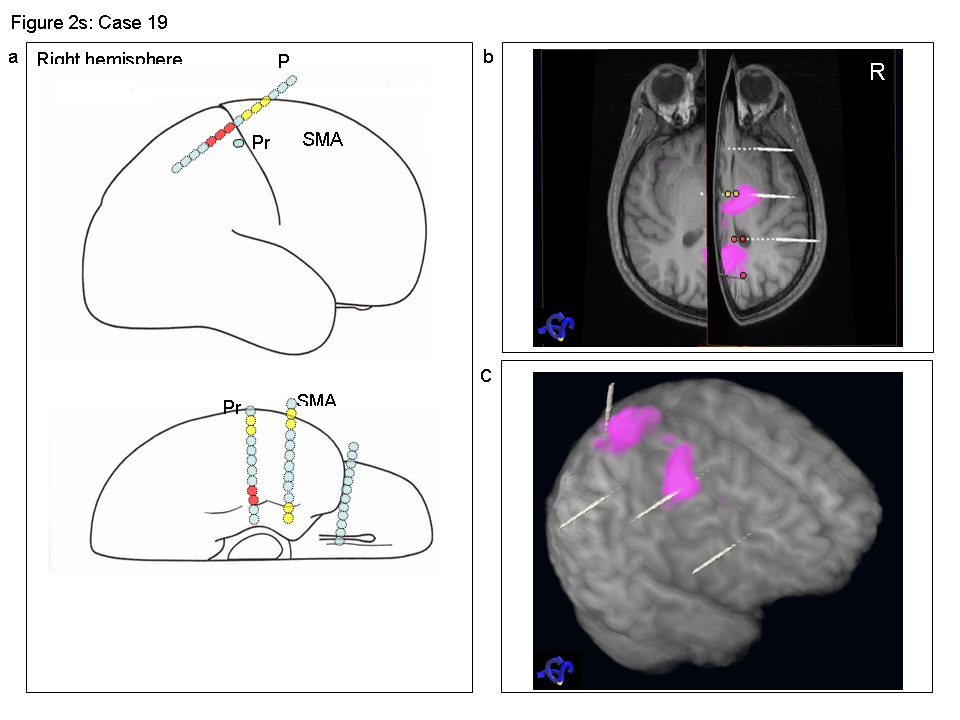

Supplement: Supplementary file 2 [file ana0070-0822-SD2.tif]

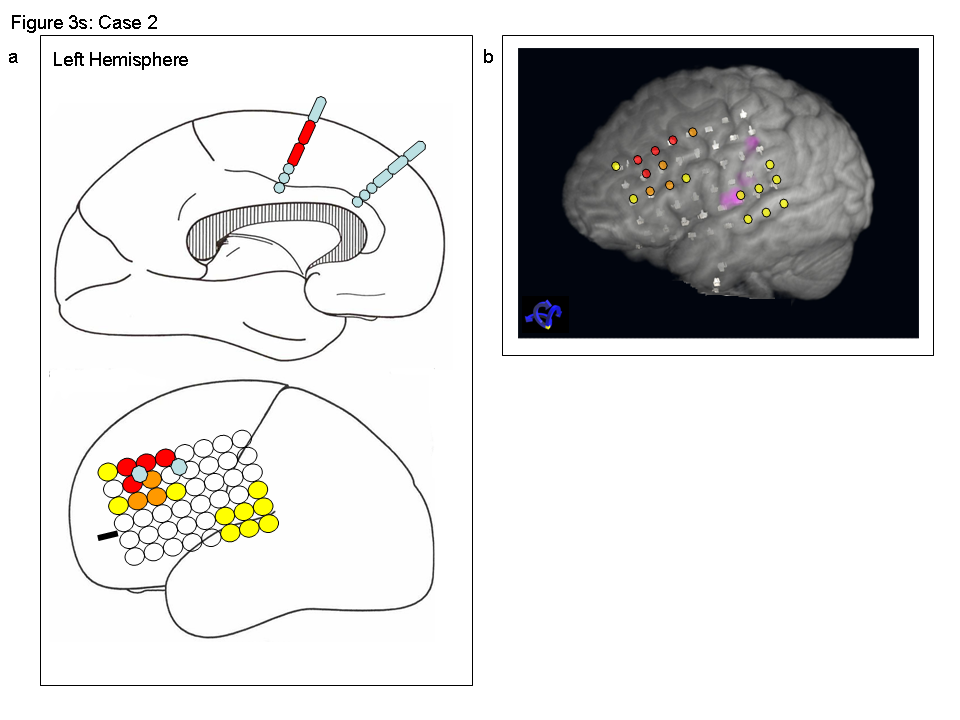

Supplement: Supplementary file 3 [file ana0070-0822-SD3.tif]

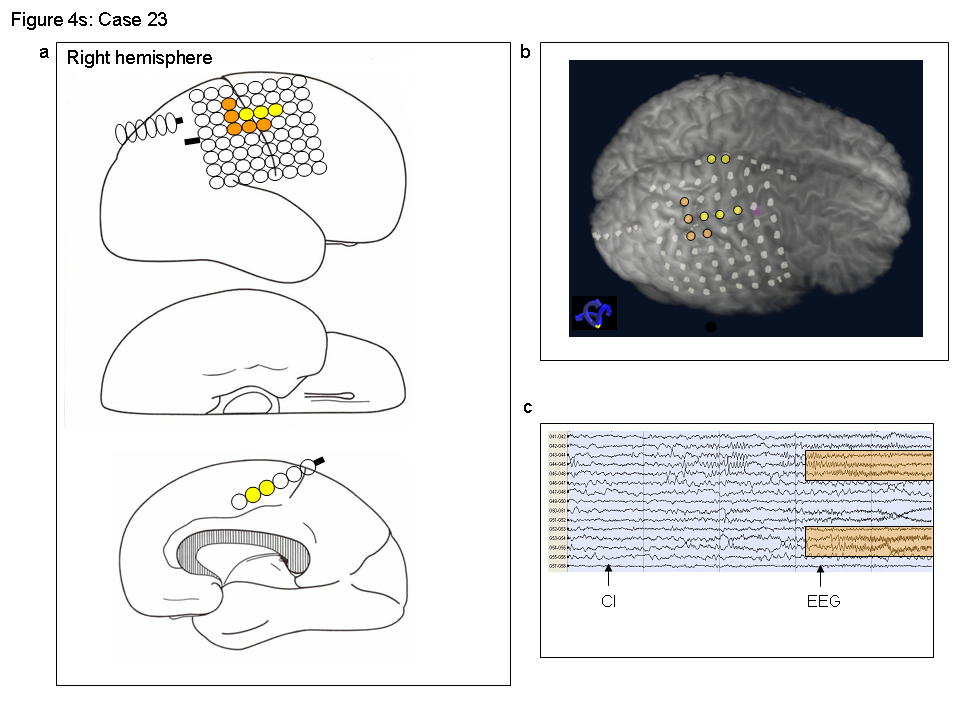

Supplement: Supplementary file 4 [file ana0070-0822-SD4.tif]
