# Supplementary material for: Epileptic Networks in Focal Cortical Dysplasia Revealed Using Electroencephalography–Functional Magnetic Resonance Imaging
Source: Ann Neurol. 2011 Dec 7;70(5):822–37. doi: 10.1002/ana.22535 (PMC3500670; doi:10.1002/ana.22535)
Supplement: Supplementary file 5 [file ana0070-0822-SD5.doc]

|  | **Site of Seizure onset zone (icEEG)** | **Implantation details** | **Seizure onset and propagation (rapid propagation = <0.5 seconds from onset)** | **Interictal discharges (IZ)** | **Resection Details** | | |
| --- | --- | --- | --- | --- | --- | --- | --- |
|  |  |  |  |  | **Type** | **Relationship to BOLD signal change** | **Size/Histology** |
| 1 | Post L SFG | 32c Gr (Gr 1-32) over left fronto-parietal convexity, 6c depth (D1-6) to lesion (sup FG) | Fast activity D4-6, Gr 11 overlying lesion. | D4-6, Gr 11,12,18,19 (cortex overlying lesion in LSFG) | None (overlap primary motor cortex) | N/A | N/A |
| 2 | Anterior L MFG | 32c Gr (Gr 1-32) over left lateral frontal convexity, 6c depth to lesion in Left MFG | Fast activity anterior grid spreading to D5-6 | Superior grid (anterior and posterior contacts) | Anterior left frontal resection | None | FCD type 2b, no clear resection margin  6x4cm |
| 3 | L middle frontal gyrus | 48c Gr (Gr1-48) over left lateral frontal convexity, 16c Str (2Gr 1-16) over left IFG and left STG. 2x depths to (DA1-6, DP1-6) | Fast activity posterior-superior quadrant of grid overlying lesion. Rapid propagation to precentral gyrus | Supero-posterior grid, superior strip overlying LIFG, lateral contacts of DA. | Left frontal coticectomy | Included cluster containing GM. Additional clusters in the same lobe | FCD type 2a, clear resection margin  3x4cm |
| 9 | RT lobe (basal-mesial) | sEEG; 10x 10c depths to right hemisphere (temporo-par-occ coverage), 1x 10c depth to left | Fast activity mesial contacts RH depth R basal T depths spreading posteriorly | RH depth (mes contacts), R basal T depth (mes contacts), RO and RP dpeths | Right anterior temporal lobe resection (partial resection of lesion as risk of visual field defect) | Included cluster containing GM. Additional extensive clusters. | FCD type 2a, no clear resection margin  2.3x3.1cm, normal hippocampus |
| 12 | L post TL and L parietal lobe (likely other regions not seen) | 32c Gr (Gr1-32) over left temporo-parietal convexity, posterior to previous resection margin. 1x frontal 8c strip (LF 1-8), 1x temporal 8c strip (LT1-8) | Fast activity over posterior-inferior quadrant of grid (Gr1-4, 9-13) propagates superior and anteriorly | Posterior half of grid and LT strip | Extension of previous resection margin | None | Gliosis following previous resection |
| 16 | L prim sensory cortex | 48c Gr (1-48) over left temporo-parietal convexity | Fast activity and spikes building up over lesion | As SOZ | Lesionectomy | N/A | FCD type 2b, fragmented |
| 18 | R medial prim sensory cortex | 64c grid over pericentral cortex. 2x 6c depths to mesial post-central gyrus (DA1-6) and mes parietal lobe (DP1-6) | Fast activity and spikes over lesion and lateral 2 contacts of anterior depth | As SOZ | Lesionectomy | Included area of max signal change | FCD type 2b, 4x 3.3 cm, clear resection margin |
| 19 | R prim sensori-motor cortex | sEEG: 3x 15c transverse electrodes (R pre-motor cortex, R pre-central gyrus, R parietal). 1x 15c oblique electrode (R medial pre and post central gyrus) | Fast activity and repetitive spikes over mesial and lateral contacts of R pre-central depth. | Medial contacts and lateral contacts R pre central, R SMA depths | Gamma knife right pre-central gyrus | Gamma knife in region of max signal change. Additional cluster in the same lobe. | Gamma knife |
| 20 | R TPO junction  L TPO junction  R frontal | sEEG: 9x 15c depth electrodes (R, T, P and O lobes) 1x15c depth electrode (LP) | Independent foci of fast activity building in right and left fusiform gyrus mesial contacts | Widespread spikes RT, RP and LP depth electrodes | None | None | N/A |
| 21 | R OL | 32c Gr R medial occipital, 16c strip R med Occ. | Fast activity building over infero-mesial contacts of L mes occ grid. Rapid spread to Right hemisphere | R mes occipital Gr c2-16 | Limited R occipital lobe resection (to avoid visual field defect) | Included area of max signal change | FCD type 2a 3x2.2cm |
| 22 | L medial OL | 20c Gr L lateral occipital, 20c Gr L temporo-parietal, 16c Str, L med occipital | Repetitive spikes and fast activity over mesial contacts lateral occ grid. Rapid propagation to R mes occ strip | L lateral occipital grid (widespread) | Left occipital lobe resection | Included area of max signal change. Additional cluster in the same lobe | FCD type 2a |
| 23 | R parietal | 64c Gr R TPF, depths to R post T (RPT 1-8) and RP (RP1-6) | Unclear | Superior Gr and RPT depth lateral contacts | None | N/A | N/A |
